# Nesting, brood rearing, and summer habitat selection by translocated greater sage‐grouse in North Dakota, USA

**DOI:** 10.1002/ece3.7228

**Published:** 2021-02-19

**Authors:** Kade D. Lazenby, Peter S. Coates, Shawn T. O’Neil, Michel T. Kohl, David K. Dahlgren

**Affiliations:** ^1^ Department of Wildland Resources Jack H. Berryman Institute S. J. Quinney College of Natural Resources Utah State University Logan UT USA; ^2^ Western Ecological Research Center Dixon Field Station U.S. Geological Survey Dixon CA USA

**Keywords:** Galliformes, greater sage‐grouse, land cover change, resource selection function, sagebrush, translocation

## Abstract

Human enterprise has led to large‐scale changes in landscapes and altered wildlife population distribution and abundance, necessitating efficient and effective conservation strategies for impacted species. Greater sage‐grouse (*Centrocercus urophasianus*; hereafter sage‐grouse) are a widespread sagebrush (*Artemisia* spp.) obligate species that has experienced population declines since the mid‐1900s resulting from habitat loss and expansion of anthropogenic features into sagebrush ecosystems. Habitat loss is especially evident in North Dakota, USA, on the northeastern fringe of sage‐grouse’ distribution, where a remnant population remains despite recent development of energy‐related infrastructure. Resource managers in this region have determined a need to augment sage‐grouse populations using translocation techniques that can be important management tools for countering species decline from range contraction. Although translocations are a common tool for wildlife management, very little research has evaluated habitat following translocation, to track individual behaviors such as habitat selection and fidelity to the release site, which can help inform habitat requirements to guide selection of future release sites. We provide an example where locations from previously released radio‐marked sage‐grouse are used in a resource selection function framework to evaluate habitat selection following translocation and identify areas of seasonal habitat to inform habitat management and potential restoration needs. We also evaluated possible changes in seasonal habitat since the late 1980s using spatial data provided by the Rangeland Analysis Platform coupled with resource selection modeling results. Our results serve as critical baseline information for habitat used by translocated individuals across life stages in this study area, and will inform future evaluations of population performance and potential for long‐term recovery.

## Graphical abstract

Decades of human enterprise have impacted populations of greater sage‐grouse (*Centrocercus urophasianus*) in the western United States. Although translocation is a viable management option to restore populations in areas where habitat has recovered, quantitative planning tools are needed to promote success of restoration efforts. We developed seasonal resource selection models for translocated sage‐grouse and evaluated habitat changes since the late 1980s to help guide future translocation decisions that will benefit population recovery.

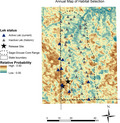

## 1 INTRODUCTION

Recent expansion of human enterprise within the American West has altered landscapes in ways that increase the risk of losing important ecosystem processes (Berquist et al., 2007; West, 1983; Whisenant, 1989). Wildlife populations, especially those of obligate species, are at particularly high risk as a result of anthropogenic landscape alterations that reduce or degrade critical habitat. Thus, there remains a need for proactive conservation actions that help to maintain functioning ecosystems. Wildlife translocations and reintroductions are a conservation action that can help maintain populations and distributions of species (Converse et al., 2013; Griffith et al., 1989; Wilson, 1988). Wildlife translocation has been described by the International Union for Conservation of Nature as the deliberate and meditated movement and release of captive or wild animals into novel and free environments (Seddon et al., 2012). Over 700 wildlife translocations of various species were documented from 1973 to 1986 across the globe extending from Australia to Hawaii, including North America (Griffith et al., 1989; Seddon et al., 2012).

Translocations may include several objectives (Converse, Moore, Folk, et al., 2013), singularly or combined, such as augmentation of declining populations, removal of nuisance animals, reintroduction of an extirpated species, establishment of a nonendemic species, and increasing genetic diversity (Gruber‐Hadden et al., 2016; Mussmann et al., 2017; Oyler‐McCance & Quinn, 2011; Smith & Clark, 1994). The objectives of many past translocations have been to re‐establish or augment extirpated or declining wildlife populations (Griffith et al., 1989; Jachowski et al., 2016; Seddon et al., 2007, 2012). Although successful translocations have been documented previously, many translocation attempts have been plagued with uncertainty and failure due to poor planning, low number of translocated individuals, and lack of essential resources (Armstrong et al., 1999; Armstrong & Craig, 1995; Converse et al., 2013; Seddon et al., 2007, 2012). Incorporating preproject planning, habitat assessments, health risk assessments, appropriate source populations, and campaigning for community support can increase the probability of success (Kleiman, 1989). These attributes were exemplified when Kleiman (1989) translocated bison (*Bison bison*) in Wichita, Oklahoma and during Baxter et al.’s (2008) successful translocation and augmentation of a greater sage‐grouse (*Centrocercus urophasianus*; hereafter sage‐grouse) population in Strawberry Valley, Utah. In addition, decision analysis, collaborative adaptive management approaches, and Bayesian population modeling approaches can help to guide decision‐making when projects are characterized by inherent uncertainty and data limitations (Converse, Moore, & Armstrong, 2013; Converse, Moore, Folk, et al., 2013; Gedir et al., 2013).

Sage‐grouse are a sagebrush obligate species that currently occupies 11 states and two Canadian provinces (Schroeder et al., 2004). Since the mid‐1900s, sage‐grouse populations have decreased by ≥33% and currently occupy ~56% of their historical distribution (Aldrich, 1963; Connelly & Braun, 1997; Connelly et al., 2011; Schroeder et al., 2004). Translocations have been used to manage sage‐grouse populations since 1933 when Allred (1946) translocated sage‐grouse into New Mexico. Since then, translocations have occurred in seven western states (Reese & Connelly, 1997). Reportedly, over 7,200 sage‐grouse have been translocated during at least 56 translocation events over the last century (Coates et al., 2006; Reese & Connelly, 1997; Snyder et al., 1999). However, the outcomes of many of these projects have been unclear due to lack of postrelease monitoring of sage‐grouse and little to no experimental study designs (Musil et al., 1993; Reese & Connelly, 1997; Snyder et al., 1999). An evaluation of past translocations has indicated that population‐level recruitment of a large number of sage‐grouse, either directly via survival of translocated individuals or indirectly via reproductive success, is needed over multiple years to achieve translocation project objectives and determine successful recovery (Baxter et al., 2008, 2013; Seddon et al., 2007, 2012; Snyder et al., 1999). In populations of other species, habitat connectivity, age structure, and number of individuals released have been identified as important for achieving desired outcomes (Lawes et al., 2013; Runge, 2013). Additional factors that correspond with success for translocated sage‐grouse include capture locations, release location attributes, and timing of release (Baxter et al., 2013; Reese & Connelly, 1997). These attributes, however, were based on observational data from the respective studies and did not come from a robust quantitative assessment. Because it has been recommended that translocations be implemented before a population declines to a level where it could be at risk of stochastic events leading to extirpation (Baxter et al., 2008), data collection characterizing individual behaviors following initial translocation will provide important information for future translocation events.

A remnant population of sage‐grouse in southwestern North Dakota represents a current and relevant example of a declining population at risk of extirpation on the northeastern fringe of their range (Garton et al., 2011; Stiver et al., 2006). Historically, sage‐grouse have been documented in the far southwest portion of the state where sagebrush (*Artemesia* spp.) was most prevalent (Johnson & Knue, 1989; Smith et al., 2004), representing part of a metapopulation that extended into southeastern Montana. Although male lek counts historically numbered in the hundreds, North Dakota's relatively small sage‐grouse population experienced consistent declines from the early 1970s to mid‐2000s. The population declined precipitously following a *West Nile virus* (WNV) outbreak in the mid to late 2000s (Walker & Naugle, 2011), and by 2016 biologists counted a total of 15 males across six active leks within the state. Although this population represents only a small portion of sage‐grouse range‐wide, the extirpation of sage‐grouse from North Dakota would be detrimental to state and range‐wide conservation objectives (Robinson, 2014). In order to address these concerns, translocation of sage‐grouse to North Dakota was proposed from a source population in south‐central Wyoming during 2017 and 2018.

Our primary study objectives were to investigate habitat selection behaviors by translocated breeding and nonbreeding female sage‐grouse and develop spatially explicit habitat maps that can indicate relative habitat use and habitat potential across life stages. Knowledge of habitat use following translocation is needed to establish minimum habitat requirements in this region, which can then be used to map distribution and guide future studies designed to infer breeding success, survival, and long‐term potential for persistence. We used a resource selection function (RSF) framework to evaluate spatial variation in habitat selection patterns for different life stages (e.g., nesting, brood rearing, and summer). A secondary objective was to examine the difference in shrub cover from 1987 to 2018 based on data provided by Rangeland Analysis Platform (RAP; https://www.climatehubs.usda.gov/hubs/southwest/tools/rangeland‐analysis‐platform) to better understand changes to sage‐grouse habitat in our study area. We hypothesized (a) that sage‐grouse selection would be closely tied to sagebrush/shrub cover and mesic habitat, and (b) that shrub cover decline between 1987 and 2018 might imply changes in habitat availability.

## 2 METHODS

### 2.1 Study area

**FIGURE 1:**
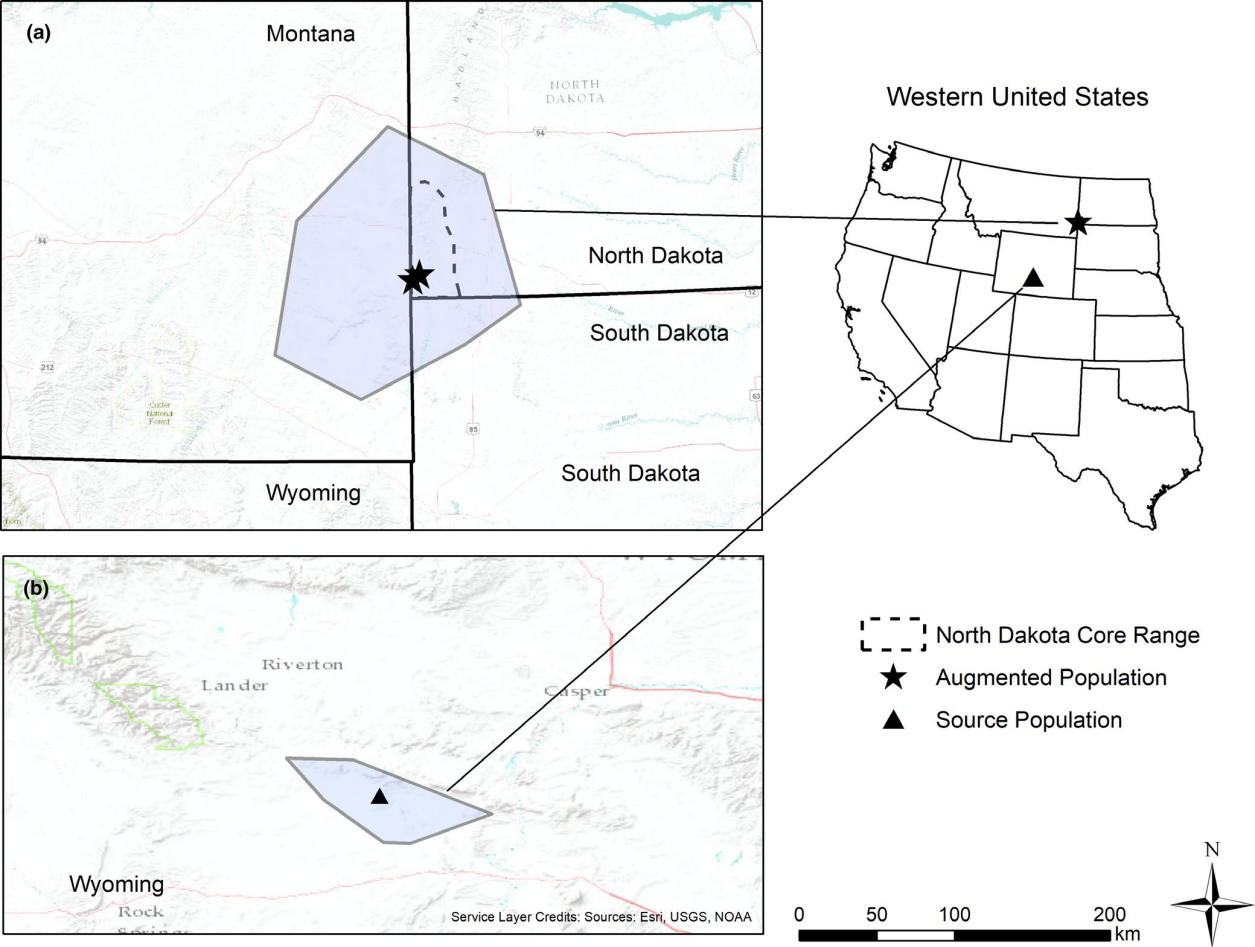
North Dakota translocation project study site locations. Our two study areas included (a) the augmented population (i.e., where Greater sage‐grouse, *Centrocercus urophasianus*, were moved to) located in Bowman and Slope Counties in southwestern North Dakota (46.050780, −104.028600) and (b) the source population (i.e., where sage‐grouse were taken from) located in the Stewart Creek Area in south‐central Wyoming (42.068902, −107.611964). Our two study boundaries were defined by minimum convex polygons based on radio‐marked sage‐grouse locations associated with the two populations, 2017–2018

Our study area incorporates two distinct study sites (Figure 1): the augmented population (i.e., where sage‐grouse were translocated to and released) in southwestern North Dakota and the source population (i.e., where sage‐grouse were captured) in south‐central Wyoming. The North Dakota study area is part of the Great Plains Sage‐Grouse Management Zone (SMZ), approximately centrally located at 46.050780, −104.028600 (Garton et al., 2011; Stiver et al., 2006). The source population occupied the Stewart Creek area in south‐central Wyoming (42.068902, −107.611964) and is part of the Wyoming Basin SMZ (Garton et al., 2011).

The Great Plains SMZ consisted of populations adjacent to North Dakota's sage‐grouse population in southeastern Montana and northwest South Dakota (Garton et al., 2011; Stiver et al., 2006). Elevation ranged from 900 to 1,052 m. Annual precipitation was 36.9 cm with a majority during the months of May and June. Average annual temperatures were 12.7 and −0.8°C (US Climate Data, 16 Oct 2018). This study site was a mixture of private, BLM, and state‐owned land. Primary land use was energy development, row crop agriculture, and livestock grazing. The landscape included gravel roads, oil pads, and power lines throughout the area. Vegetation in this area was on the edge of the shrub‐steppe and shortgrass prairie bioregions. A patchwork of shrub‐steppe habitats included a mixture of shrub species, often dominated by sagebrush, with an understory of perennial and annual forbs and grasses, as well as vast areas of open grasslands (Johnson & Larson, 1999). Within shrub‐steppe communities, shrub species included silver sagebrush (*A. cana*), big sagebrush (*A*. *tridentata*), western snowberry (*Symphoricarpos occidentalis*), rubber rabbitbrush (*Chrysothamnus nauseosus*), and greasewood (*Sarcobatus vermiculatus*) (Johnson & Larson, 1999). The dominant grasses consisted of Kentucky blue grass (*Poa pratensis*), western wheatgrass (*Pascopyrum smithii*), Japanese brome (*Bromus japonicas*), needle and thread (*Stipa comada*), and June grass (*Koeleria macrantha*). Prevalent forbs were common yarrow (*Achillea millefollium*), common dandelion (*Taraxacum officinale*), and textile onion (*Allim textile*) (Johnson & Larson, 1999).

The translocation source population was located in Carbon and Sweetwater counties in south‐central Wyoming. At this site, elevation ranged from 1,520 to 2,080 m and experienced average annual precipitation of 23.5 cm and temperatures ranged from 13.0 and −1.5°C (US Climate Data, 16 Oct 2018). Private lands were interspersed with public lands that were primarily administered by federal and state agencies. Managed livestock grazing, in the form of domestic sheep and cattle, was the dominant land use. Sagebrush vegetation communities dominated the majority of the study area. Wyoming big sagebrush (*A. tridentata wyomingensis*) and mountain big sagebrush (*A. t. vaseyana*) were the most common. Black sagebrush (*A. nova*) and dwarf sagebrush (*A. arbuscula*) were found on exposed ridges. Other common shrub species at this site included: antelope bitterbrush (*Purshia tridentata*), Common snowberry (*Symphoricarpos albus*), chokecherry (*Prunus virginiana*), alderleaf mountain mahogany (*Cercocarpus montanus*), rabbitbrush (*Chrysothamnus* and *Ericameria* spp.), greasewood, saskatoon serviceberry (*Amelanchier alnifolia*), and spiny hopsage (*Grayia spinosa*). Isolated stands of juniper (*Juniperus* spp.) and quaking aspen (*Populus tremuloides*) were found at the higher elevations on north‐facing hillsides.

### 2.2 Capture and marking

We captured sage‐grouse at night using spotlight and dip‐net methods (Connelly, 2003; Giesen et al., 1982; Wakkinen, Reese, & Connelly, 1992; Wakkinen, Reese, Connelly, & Fischer, 1992) from all‐terrain vehicles. Upon capture, sage‐grouse were sexed, aged, and fitted with aluminum leg band with unique identification numbers (Braun & Schroeder, 2015; Eng, 1955; Patterson, 1952). Female sage‐grouse were also fitted with either rump‐mounted solar‐powered GeoTrak Global Positioning System‐Platform Transmitter Terminal (GPS‐PTT; hereafter GPS) transmitters (~30 g) with a 3.5 g very high frequency (VHF) radio (Holohil Systems, Ltd.) epoxied to the side of the GPS transmitter, or Advanced Telemetry Systems or Holohil Systems, Ltd. VHF necklace style transmitters (22 g).

While some sage‐grouse were captured and translocated, we also captured and marked another group of female sage‐grouse that were fitted with the same units and then immediately released back into the source population. The purpose of this effort was to monitor the source population and plan for the translocation of broods (i.e., adult female and chicks) from females that successfully hatched within the source population in 2018.

Using nocturnal spotlight methods described above, we recaptured previously marked females and their chicks in June and July 2018. Following handling procedures after capture, the brood female and chicks were placed in the translocation release boxes for transport and then were released in North Dakota.

We monitored all radio‐marked female sage‐grouse either remotely via ARGOS‐enabled downloads (https://www.argos‐system.org/) or with ground telemetry using VHF signals. We only used ground telemetry to approach within a few meters of marked females to verify nest initiation and to monitor nest status. Marked brood females with chicks were monitored remotely until recaptured and the entire brood was translocated. Veterinarians from Wyoming Game and Fish Department and North Dakota Game and Fish Department (NDGF) attended all spring translocations and a local veterinarian attended the brood captures. Veterinarians examined the general health of all translocated sage‐grouse and obtained blood and swab samples from translocated sage‐grouse for disease testing. Colorado State University Veterinary Diagnostic Laboratory tested for *Mycoplasma*, and Michigan State Veterinary Laboratory tested for *Salmonella* and *Mycobacterium Avium*, and Wyoming State Veterinary Laboratory tested for *Avian Influenza*. An agreement with North Dakota State Board of Animal Health indicated that translocated grouse could be released in North Dakota as quickly as possible prior to researchers receiving disease testing results to avoid holding the birds for an inordinate amount of time, with the caveat that if any results were positive then those individuals would be immediately located via telemetry and humanely euthanized.

### 2.3 Translocation and release methods

Sage‐grouse were translocated either in a fixed‐wing aircraft provided by NDGF or a covered truck to the release locations within the augmented population. Prior to release, each bird translocated in spring was transferred into an individual compartment within a manufactured release box fixed with a remote door opener to enable a soft release (Rodgers, 1992). On the morning of release, we placed all translocated sage‐grouse near predetermined lek sites (i.e., “pseudo‐leks”), constructed silhouette decoys, and used Fox pro NX4s (FOXPRO Inc.) to transmit prerecorded sage‐grouse lekking sounds, in an attempt to decrease postrelease stress and movements (Baxter et al., 2008; Coates et al., 2006; Snyder et al., 1999). All translocated broods were transported via truck using a specially made brood box (~8 hr), released into acclimation pens (~30 to 45 min), and then released into nearby sagebrush. Brood augmentation sites were also distributed close to historical lek sites near initial spring translocations. When identifying release locations, we also considered brooding areas where endemic broods had been detected, along with the availability of sagebrush and mesic habitat (Connelly et al., 2011; Dahlgren et al., 2010). All capture and handling procedures were approved by The Utah State University Institutional Animal Care and Use Committee (IACUC; permit #11079).

### 2.4 Data collection

All sage‐grouse were monitored intensely during the nesting and brooding seasons (April–August) to identify nesting and brooding activity. GPS locations were downloaded remotely via ARGOS‐enabled downloads (http://www.argos‐system.org/) from transmitters that were programmed with 4 seasons, which were as follows: March–May, May–June, June–October, and October–March. Up to six locations were gathered per day for all seasons. We also used ground telemetry for VHF signals and approached within a few meters of marked females to verify nest initiation, nest success, brooding activity, or mortality. Females marked with VHF transmitters were located weekly or as often as possible with handheld Communication Specialist R1000 receivers (Communication Specialist Inc.), and Yagi antennae. We located all nesting and brooding females at least once per week. We completed nocturnal spotlight brood checks at roost locations 20, 30, and 50 days posthatch (Dahlgren et al., 2010). We also used pointing dogs to locate broods under rare circumstances when we were unable to locate broods at night for broods greater than 30 days post hatch, or if the VHF transmitter failed on GPS transmitters (Dahlgren et al., 2010). Following analysis, resource selection model results used for this manuscript were published as a USGS data release (Coates et al., 2021).

### 2.5 Resource selection analysis

#### 2.5.1 Landscape variables

Landscape variables were selected based on biological significance to sage‐grouse habitat use. We categorized variables into topographic, biological, and anthropogenic factors (Connelly et al., 2000, 2011; Table 1). Topographic variables included elevation, slope, ruggedness (Riley et al., 1999), and aspect. These variables were derived from 30‐m DEM (https://viewer.nationalmap.gov/advanced‐viewer, accessed 1 October 2018). Biological factors included linear water (e.g., rivers and streams) from US census database (https://tigerweb.geo.census.gov/tigerweb, accessed October 1, 2018) mesic habitat, and percent shrub canopy cover from RAP (https://rangelands.app/, accessed June 11, 2019). State and federal roads were also acquired from US census database and used to characterize anthropogenic effects.

**TABLE 1:** Landscape variables that were selected for analysis based on biological significance to greater sage‐grouse (*Centrocercus urophasianus*) habitat use

Variable	Category	Description/Source	References
Elevation	Topographic	Extracted from 30 m DEM	Coates, Casazza, et al. (2016), Gibson et al. (2016), Gibson et al. (2018), O’Neil et al. (2020)
Aspect	Topographic	Extracted from 30 m DEM	Gibson et al. (2016), Gibson et al. (2018)
Slope	Topographic	Extracted from 30 m DEM	Gibson et al. (2016), Gibson et al. (2018)
Ruggedness^a^	Topographic	Calculated from 30 m DEM	Doherty et al. (2008), Fedy et al. (2014), Coates, Casazza, et al. (2016), Walker et al. (2016), O’Neil et al. (2020)
Distance to Road	Anthropogenic	U.S. Census Bureau	Fedy et al. (2014), Gibson et al. (2016), LeBeau et al. (2017)
Distance to Water	Biological	U.S. Census Bureau	Casazza et al. (2011), Connelly et al. (2011), Fedy et al. (2014), Coates, Casazza et al. (2016)
Distance to Mesic area	Biological	Polygons layers provided by Sage‐grouse Initiative (SGI)	Hagen et al. (2007), Casazza et al. (2011), Fedy et al. (2014), Coates et al. (2020)
Shrub Cover^a^	Biological	Percent shrub cover of years of interest provided by Rangeland Assessment Platform	Aldridge et al. (2008), Connelly et al. (2011), Fedy et al. (2014), Coates, Casazza, et al. (2016), Doherty et al. (2016), Walker et al. (2016)

Note

We categorized landscape variables into topographic, biological, and anthropogenic factors. Topographic variables included elevation, slope, ruggedness, and aspect. These variables were derived from a 30‐m digital elevation model (DEM). Biological factors included rivers, streams, springs, mesic, and shrub cover from the Rangeland Analysis Platform (https://rangelands.app). Anthropogenic variables include state and federal roads derived from the U.S. Census Bureau database Tiger/Line files (https://tigerweb.geo.census.gov/tigerweb).

^a^ Multi‐scale representations considered: circular moving window radii of 60, 331, and 887 m, respectively, for nesting and brood‐rearing seasons, and 111, 767, 1,503, and 3,005 m respectively, for summer, nonbreeding season.

We estimated distance metrics for roads, water, and mesic variables in ArcMap 10.3 (ESRI; Dinkins et al., 2014; Knick & Connelly, 2011; Sandford et al., 2017; Wisdom et al., 2011). To account for spatial dependence associated with habitat selection of sites near leks at the release location (Coates et al., 2013), we included distance to release location pseudo‐lek as an additional predictor in our models. This variable was important to capture habitat selection patterns without confounding estimated effects of other environmental factors, because we expected sage‐grouse to select habitats and/or establish home ranges near the release site initially, with possibly diminishing effects over time. Thus, applying an RSF framework to individuals that are spatially associated with habitat‐independent locations (such as leks) requires accounting for these features to avoid biases and misinterpretation of the RSF coefficients (e.g., Gibson et al., 2016). In addition, because sage‐grouse have demonstrated selection of landscape features at different spatial scales across seasons (Connelly et al., 2011; Fedy et al., 2014; McGarigal et al., 2016), we evaluated log‐transformed ruggedness and shrub canopy cover using a circular moving window (focal statistics neighborhood analysis). The size of the moving window was controlled by a variable radius chosen to represent daily movement patterns (e.g., Coates, Brussee, et al., 2016; Coates, Casazza, et al., 2016; Fedy et al., 2012; Holloran & Anderson, 2005), measured by the average minimum, mean, and maximum movements from all individuals during nesting and brood rearing (*r* = 60, 331, and 887 m, respectively), as well as separate estimates for summer (*r* = 111, 767, and 3,005 m, respectively). Due to the wide range of movements during summer, we considered an additional radius length (*r* = 1,503 m) that represented half the maximum movement. We did not evaluate scale‐dependent responses with respect to other continuous variables, because averaging within the moving window might obscure local‐level information (e.g., aspect) and because broader scale variation in topography (e.g., elevation and slope) was already captured by multi‐scale analysis of topographic roughness. To accommodate interannual variability, we used percent shrub canopy cover obtained from RAP, with data estimated annually for 1987, 2005–2009, and 2017–2018. We associated the percent shrub canopy cover data with the respective years throughout our study site.

For point and linear features (center of mesic area, water bodies or streams, roads, and pseudo‐lek release locations), we calculated exponential decay functions, exp(−*d*/α), to accommodate declining effect sizes with increasing distance (Coates, Brussee, et al., 2016; Coates, Casazza, et al., 2016; Nielsen et al., 2009), where *d* represented distance to the feature, and α was specified as either the mean distance value at all used locations, or 6.4 km (e.g., Green et al., 2017), whichever was smaller. We tested for correlation using Pearson's correlation test with an |*r*| > .7 threshold for location data (Hosmer & Lemeshow, 2013). We removed slope, due to its collinear relationship with ruggedness and elevation; the latter variables were retained due to their importance to sage‐grouse habitat selection patterns in other regions (e.g., Coates, Brussee, et al., 2016; Coates, Casazza, et al., 2016; Coates et al., 2020). No other significant correlations occurred among the variables we considered.

#### 2.5.2 Nesting RSF

To increase our sample size, we combined our 2017 and 2018 nest location data with nest location data acquired during the 2005–2009 nesting seasons recorded within our same augmentation study area (Herman‐Brunson et al., 2009). Then, we created a database of used and available points within the study area using a random sampling approach to evenly sample across the study area (Benson et al., 2015), where five random points were generated for each used point to create a second order RSF (Johnson et al., 2006). The choice of a 5:1 ratio was designed to adequately cover the study area (Northrup et al., 2013) while avoiding oversampling that could contribute to spatial dependence in model residuals and underestimation of effect uncertainty. After combining used and available locations with habitat predictors, we standardized predictors (*µ* = 0, *σ* = 1) so that all were scaled similarly and coefficients were comparable among variables occurring in models.

We estimated a nest RSF using a generalized linear model within a Bayesian modeling framework. The nest model was initially estimated as follows with *g*(*x*) estimated for the *i*th pixel location where *β*_0_ is the mean intercept, *x*_1…_ *_k_* are covariates of length *k* with fixed regression coefficient *β_k_*, expressed as

$$g\left(x\right)=\beta_{0}+\beta_{1}x_{1i}+\beta_{2}x_{2i}+\cdots +\beta_{k}x_{\mathrm{ki}}$$

The observations in this model followed a *Bernoulli* distribution, where *y* = 1 indicated a nest location and *y* = 0 indicated a random background location, thereby specified as a logistic regression to obtain parameter estimates for all fixed coefficients **β** (Johnson et al., 2006; McDonald, 2013). To estimate the RSF from this model, $\hat{w}\left(x\right),$ we discarded the intercept and calculated the exponential function (Johnson et al., 2006; McDonald, 2013), which took the form:

$$\hat{w}\left(x\right)=\exp\left({\hat{\beta }}_{1}x_{1}+{\hat{\beta }}_{2}x_{2}+\cdots +{\hat{\beta }}_{k}x_{k}\right)$$

To obtain a final RSF model for habitat mapping, we performed a 2‐stage modeling process, where an appropriate spatial scale was determined for shrub cover and ruggedness during the first stage, and the model was refit to the selected spatial scales in the second stage. We selected the most informative spatial scale for these two variables using Bayesian latent indicator scale selection (Stuber et al., 2017), which employs a reversible‐jump MCMC sampling algorithm to estimate probabilities for each specified spatial scale being the most important predictor of habitat selection among those considered. We then used only the scale with the highest probability for the final RSF model.

We fit these models on ~2/3 of the dataset (e.g., training data), randomly selecting and withholding ~1/3 of the data for validation (testing data). While discrimination methods such as area under the receiver operating curve (or AUC; Peterson et al., 2008) are often applied to species distribution models to evaluate a model's ability to distinguish between used and available locations, we were primarily interested in our model's ability to predict the relative probability of occurrence (e.g., the RSF) when applied to new data (Boyce et al., 2002). Given that the RSF relies on “presence‐only” data and thereby estimates relative use as a weighted function of availability (Aarts et al., 2013; McDonald, 2013), the background availability locations cannot be assumed to represent true absence. This implies that discrimination methods may have limited utility when applied to RSFs, and validation methods that evaluate predicted intensity of locations across different habitat types likely have greater potential to characterize model performance and identify deficiencies (Boyce et al., 2002; Fieberg et al., 2018; Johnson et al., 2006). Hence, to validate the model with the testing data, we used calibration plots described in Johnson et al. (2006) and Fieberg et al. (2018) to compare the true number of observations to predicted numbers of locations occurring within 10 quantile bins from $\hat{w}\left(x\right)$ which was fit only to the training data. We report the slope and *R* ^2^ of a linear regression model fit to the calibration plot (Johnson et al., 2006), as well as the Spearman correlation, to determine how well the model predicted habitat selection patterns for individuals that were not included in the model‐fitting process. If results were not satisfactory, we generated used‐habitat calibration (UHC) plots (Fieberg et al., 2018) for predictors in the model to determine the need for nonlinear functions or interactions. If this was the case, we first fit quadratic terms to the most influential predictor and measured improvement based on the calibration statistics (e.g., improved correlation, *R* ^2^, and slope coefficient near 1.0; Johnson et al., 2006). If necessary, we continued this procedure with additional predictors until correlation and *R* ^2^ were >.75 and slope was between 0.8 and 1.2.

All models were fit using JAGS 4.2.0 (Plummer, 2003), implemented within R (R Core Team, 2019, version 3.6.1) using “rjags” (Plummer, 2016) and “jagsUI” (Kellner, 2019). To protect against potentially overfitting the model to small effective sample sizes (i.e., number of used locations), we implemented *L* − 1 regularization (Tibshirani et al., 2012) by specifying Lasso (i.e., Laplace, or double‐exponential) prior distributions for each predictor variable in the full model, with an uninformative hyperprior specified for the tuning parameter *λ* (Hooten & Hobbs, 2015; Park and Casella, 2008). Because nests were included from resident females during 2005 and 2007, in addition to translocated females, we performed a post hoc analysis contrasting habitat selection coefficients between prior residents and translocated females. Specifically, we fit an alternative model where the most influential habitat coefficients, evidenced by 85% or greater posterior probability of *β* greater or less than 0, were specified to vary by resident/translocated status. To test for any differences, we recorded the probability that *β*_resident_ > *β*_translocated_, where large (>0.95) or small (<0.05) probabilities indicated strong evidence of difference.

For all models, we ran three chains of 30,000 MCMC iterations, following a burn‐in of 15,000, and thinned by selecting every 5th sample to posterior distributions of parameter estimates. We examined chains and calculated Gelman–Rubin statistics (*r̂* < 1.05) to verify convergence of all parameters.

#### 2.5.3 Brooding RSF

We created a location database that included all brooding female sage‐grouse with ≥5 locations in 2017–2018. We selected 1 brood location per day, per individual, if multiple locations were gathered in the same day. We used similar methods as for the nest model to develop our data and run analyses, generating five random points per used location, and withholding ~1/3 of the broods as a validation dataset. One exception was that individual was treated as a random effect, to account for repeated locations gathered from the same sampling unit over time. The brood model was calculated as follows with *g*(*x*) estimated for location *i* and brood *j* where $\beta_{0}$ is the mean intercept, $x_{1\cdots k}$ are covariates of length *k* with fixed regression coefficient $\beta_{k}$, and $\gamma_{j}$ is a random intercept for brood *j*, that took the form:

$$g\left(x\right)=\beta_{0}+\beta_{1}x_{1ij}+\beta_{2}x_{2ij}+\cdots +\beta_{k}x_{\mathrm{kij}}+\gamma_{j}$$

We discarded the intercepts and calculated the exponential RSF following the same formulation for ${\hat{w}\left(x\right)}_{\mathrm{brood}}$ as was done for the nest model, this time fit to the brood location data. All other procedures followed those of the nest model.

#### 2.5.4 Summer RSF

All GPS marked female sage‐grouse locations from 2017 to 2018 were combined into an inclusive database. We then created a subset that included all nonreproductive female sage‐grouse from June through October. We removed any individual that did not provide at least 10 independent locations. We randomly selected one location per day, per individual to avoid spatial dependence between locations collected close to each other in space and time. Locations occurring on separate days were considered independent. The summer model was estimated for location *i* and individual *j* following the equation above, where $\beta_{0}$ is the mean intercept, $x_{1\cdots k}$ are covariates of length *k* with fixed regression coefficient $\beta_{k}$, and $\gamma_{j}$ is a random intercept for individual *j*. We discarded the intercepts and calculated the exponential RSF following the same formulation for $\hat{w}{\left(x\right)}_{\text{summer}}$ as was done for the nest and brood models, this time fit to the GPS location data. All other model procedures followed those of the previous models. When making spatially explicit predictions across all life stages, we omitted the release site predictor variable, as it was considered to be a nuisance parameter independent of habitat suitability.

#### 2.5.5 Annual RSF

After RSFs were generated for each season, we projected relative probability of selection surfaces across the study region for each season (nesting, brood, and summer) by applying a logistic transformation to each $\hat{w}\left(x\right)$. We then calculated the geometric mean of the three seasonal RSFs to generate an annual composite RSF to represent habitat across life stages across the study area. This surface was treated as a relative index of selection, to identify baseline habitat requirements across seasons.

#### 2.5.6 Shrub canopy cover change

For each season, as in above, we applied a logistic transformation to each $\hat{w}\left(x\right)$ (nesting, brood, and summer) based on current shrub conditions from the 2018 shrub raster layer, which was smoothed at the selected spatial scale indicated by the analyses. Then, we performed the same calculation, this time using the 1987 percent shrub canopy raster layer provided by RAP, and again smoothed at the selected spatial scale for each life stage. We subtracted the coinciding 1987 RSF surface from the 2018 RSF surface for each season, respectively. This retrospective analysis should not be directly interpreted as a change in habitat selection, because data from 1987 were not available to model habitat functional responses (Mysterud & Ims, 1998; Matthiopoulos et al., 2011). However, the results demonstrate how habitat availability has likely changed with changing shrub canopy cover over the previous 30 years within our study area by accounting for current sage‐grouse habitat selection patterns that are a function of multiple landscape variables.

## 3 RESULTS

We translocated 66 female sage‐grouse during lekking (March–April) and brooding seasons (May–July) of 2017 and 2018, from the source population site to southwestern North Dakota. During the 2017 spring breeding season, *n* = 40 female sage‐grouse were translocated. In the spring of 2018, *n* = 20 female sage‐grouse were translocated to North Dakota. During the 2018 brooding season, we translocated *n* = 6 additional females with broods (*n* = 26 chicks).

### 3.1 Nesting RSF

**FIGURE 2:**
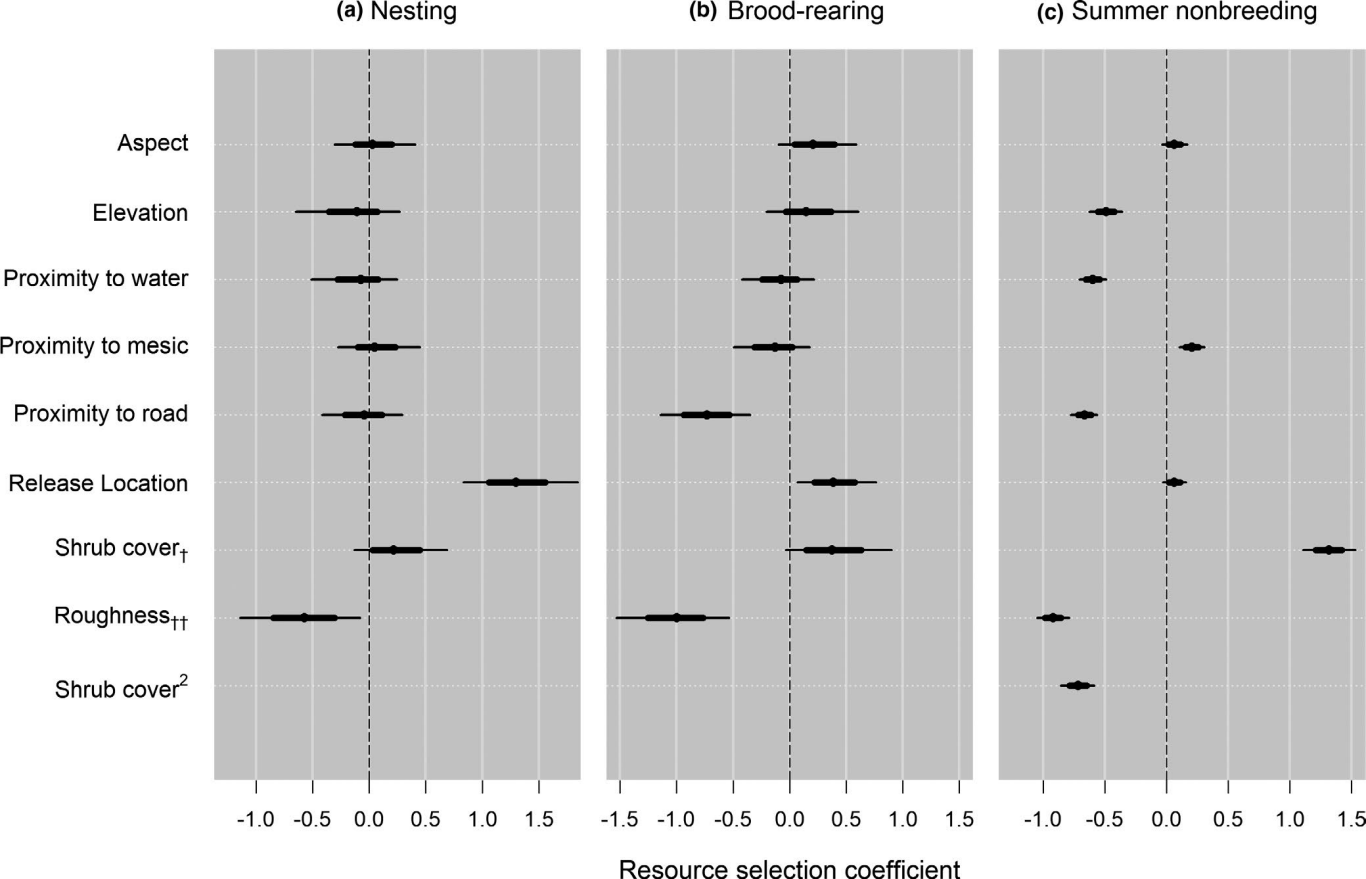
Posterior distributions of coefficients for (a) nesting, (b) brood rearing, and (c) summer nonbreeding resource selection by Greater sage‐grouse (*Centrocercus urophasianus*) females in southwestern North Dakota, USA. Plots show midpoint location, 68% credible interval, and 95% credible of each posterior distribution, represented by dot, thick line, and thin line, respectively. ^†^Shrub cover scale of selection corresponds to 887 m for nesting and brood rearing, and 1,503 m radii for summer nonbreeding resource selection, respectively. ^††^Topographic roughness scale of selection corresponds to 887 m for nesting and brood rearing, and 767 m radii, for summer nonbreeding resource selection, respectively

**FIGURE 3:**
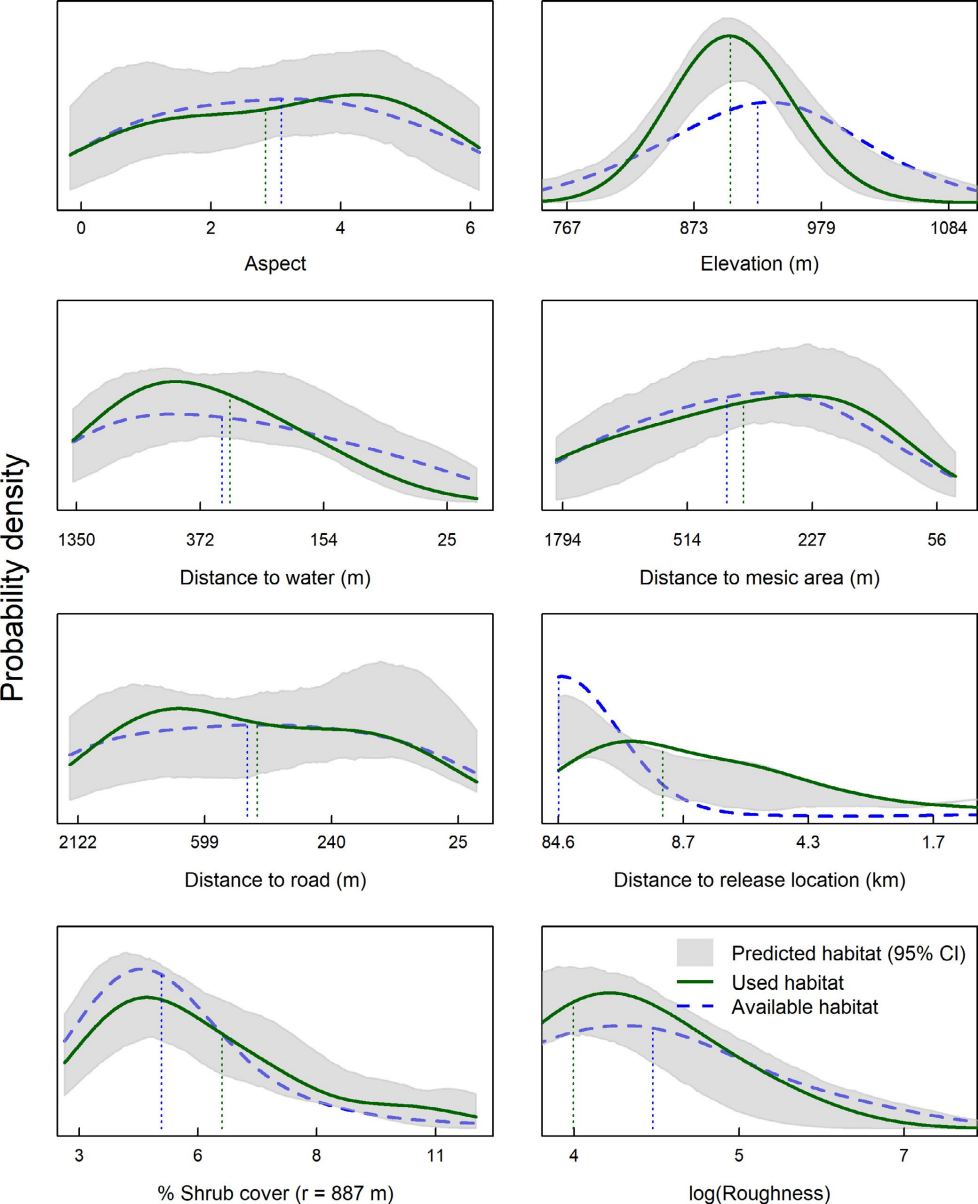
Used‐habitat calibration plots of a resource selection function of female Greater sage‐grouse (*Centrocercus urophasianus*) nest locations in North Dakota, USA, 2005–2008 and 2017–2018. Solid green lines indicate the approximate probability distribution of use for each habitat characteristic, blue dashed lines indicate distribution of habitat at random, available locations, and gray shaded areas indicate model predictions of the distribution of use at independent validation locations

We used locations from *n* = 17 nests in 2005, and *n* = 27 in 2007 from previous research. We documented new nest locations of *n* = 8 in 2017, and *n* = 9 in 2018, for *n* = 61 total nests. Our nest RSF model included elevation, roughness, aspect, distance to roads, distance to water, distance to mesic, distance to translocation release site, and percent shrub canopy cover (Figure 2). Shrub canopy cover and roughness both had greatest support for inclusion in this model when measured within an 887 m radius circular moving window (Appendix Table A1). Nonlinear functions and/or quadratic terms were deemed unnecessary based on satisfactory results from UHC plots (Figure 3). Nesting females strongly selected for nesting habitat close to the translocation release site (which occurred near historical lek locations), with lower topographic roughness and greater shrub cover relative to available locations (Table A2; Figure 3). The model predicted validation data adequately (nest locations withheld from analysis; *r* = 0.92, *R* ^2^ = 0.87, β = 0.90). We did not detect strong differences in nest habitat selection between prior year (2005, 2007) resident females and translocated females (2017, 2018), where *P*(*β*_resident_ > *β*_translocated_) values were 0.12, 0.67, and 0.52 for proximity to release site, shrub cover, and topographic roughness, respectively.

### 3.2 Brooding RSF

**FIGURE 4:**
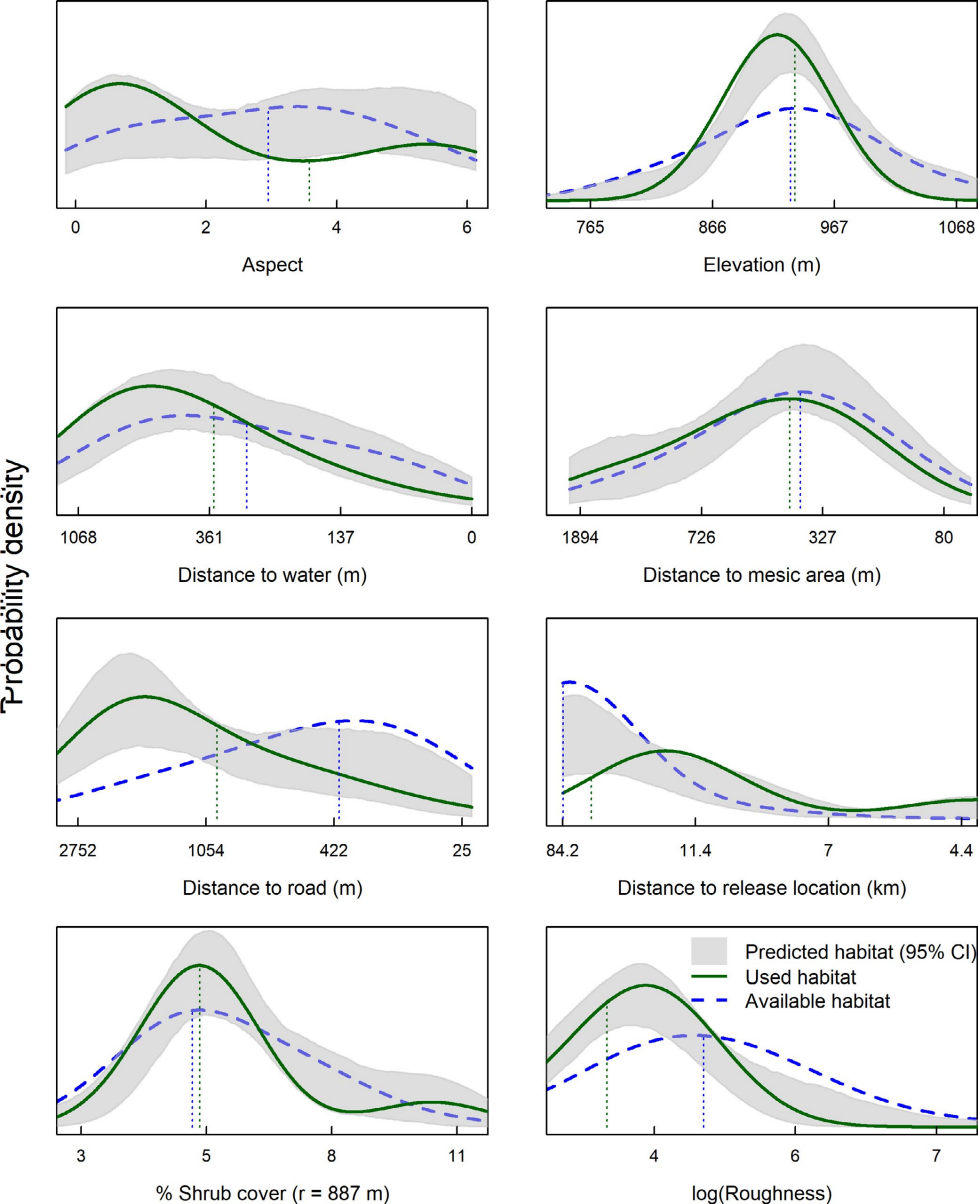
Used‐habitat calibration plots of a resource selection function of female Greater sage‐grouse (*Centrocercus urophasianus*) brood locations in North Dakota, USA, 2017–2018. Solid green lines indicate the approximate probability distribution of use for each habitat characteristic, blue dashed lines indicate distribution of habitat at random, available locations, and gray shaded areas indicate model predictions of the distribution of use at independent validation locations. The *x*‐axis indicates increasing proximity (i.e., decreasing distance from left to right) which were represented by exponential decay functions, and increasing values of continuous variables otherwise. All variables are plotted across the 2.5th to 97.5th percentile of all used and available locations

We monitored *n* = 6 broods produced by spring translocated females, and we translocated an additional *n* = 6 broods that were included in analyses. Our brood model included elevation, roughness, aspect, distance to roads, distance to water, distance to mesic, distance to translocation release site, and percent shrub canopy cover (Figure 2). Shrub canopy cover and roughness had greatest support for inclusion in this model when measured within an 887 m radius circular moving window (Table A1). Nonlinear functions and/or quadratic terms were again deemed unnecessary based on results from UHC plots (Figure 4). Relative to available locations, brooding female sage‐grouse in our augmented study area selected for areas farther from roads and mesic habitats, in closer proximity to release sites, with lower topographic roughness and greater shrub canopy cover (Table A2; Figure 4). Sage‐grouse displayed little to no selection regarding other geographic variables. The model incorporated 54 locations and predicted validation data adequately (brood locations withheld from analysis, *n* = 28; *r = 0.80, R^2^ = 0.83, β = 1.00*).

### 3.3 Summer RSF

**FIGURE 5:**
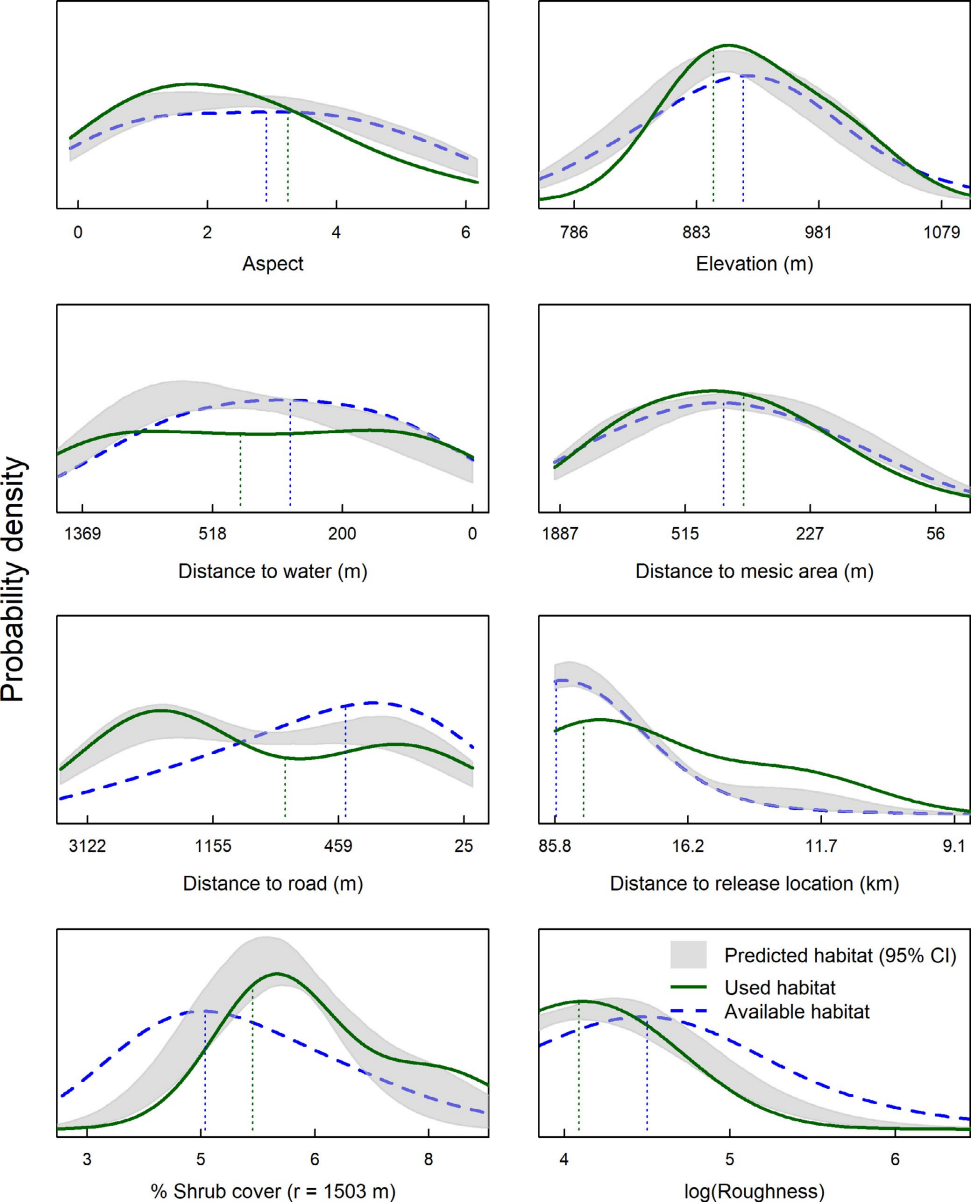
Used‐habitat calibration plots of a resource selection function of female Greater sage‐grouse (*Centrocercus urophasianus*) nonbreeding summer locations in North Dakota, USA, 2017–2018. Solid green lines indicate the approximate probability distribution of use for each habitat characteristic, blue dashed lines indicate distribution of habitat at random, available locations, and gray shaded areas indicate model predictions of the distribution of use at independent validation locations

We monitored *n* = 32 female sage‐grouse that were marked with GPS transmitters during 2017 and 2018. Our top model for summer included elevation, roughness, aspect, distance to roads, distance to water, distance to mesic, and percent shrub canopy cover (Table A2; Figure 2). We also included a quadratic term for percent shrub canopy cover, to calibrate the model based on a nonlinear relationship between habitat used and shrub cover, which improved the calibration plots (Figure 5). Shrub canopy cover had greatest support for inclusion in this model when measured within a 1,503 m radius circular moving window (*p > *.99), whereas roughness was most supported using a 767 m radius (*p* > .99). Our RSF model indicated selection relative to availability for lower relative elevations near mesic areas but further from open water sources and roads. In contrast to nesting and brooding periods, we observed little influence of translocation release sites. Shrub cover was selected at intermediate shrub canopies (quadratic effect). The model incorporated 527 locations and predicted validation data adequately (summer locations from 5 females withheld from analysis; *r* = 0.80, *R* ^2^ = 0.79, β = 1.12).

### 3.4 Seasonal change indices and annual RSF

**FIGURE 6:**
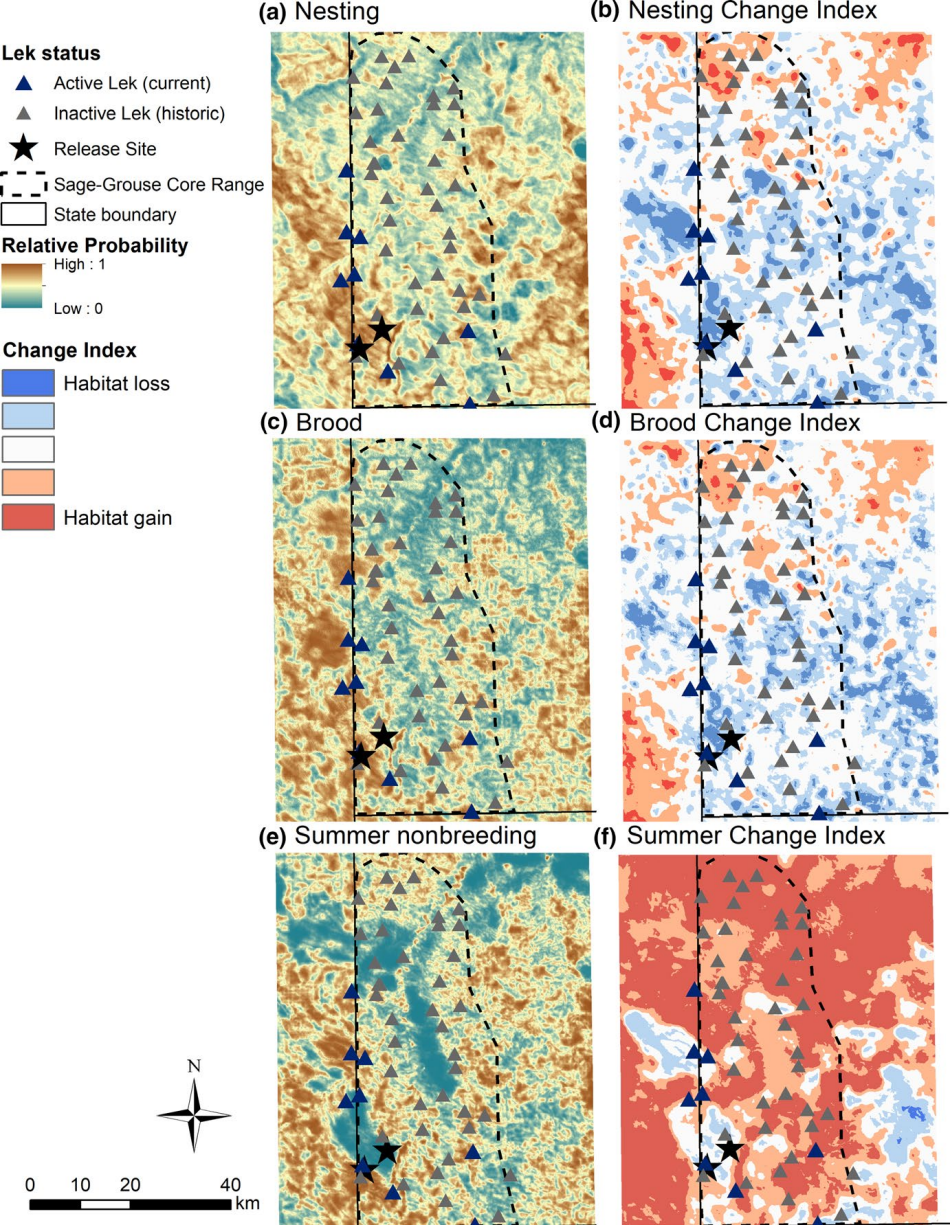
Predicted Greater sage‐grouse (*Centrocercus urophasianus*) habitat selection during 2005–2008 and 2017–2018 in southwestern North Dakota (a, c, e), and change in sage‐grouse predicted habitat between 1987 and 2018 (b, d, f) corresponding to nesting (a–b), brood rearing (c–d), and summer nonbreeding (e–f) life stages. Predictions were obtained from a resource selection function of sage‐grouse nest locations, where warmer color shades indicate greater relative probability of selection compared to cool color shaded areas

**FIGURE 7:**
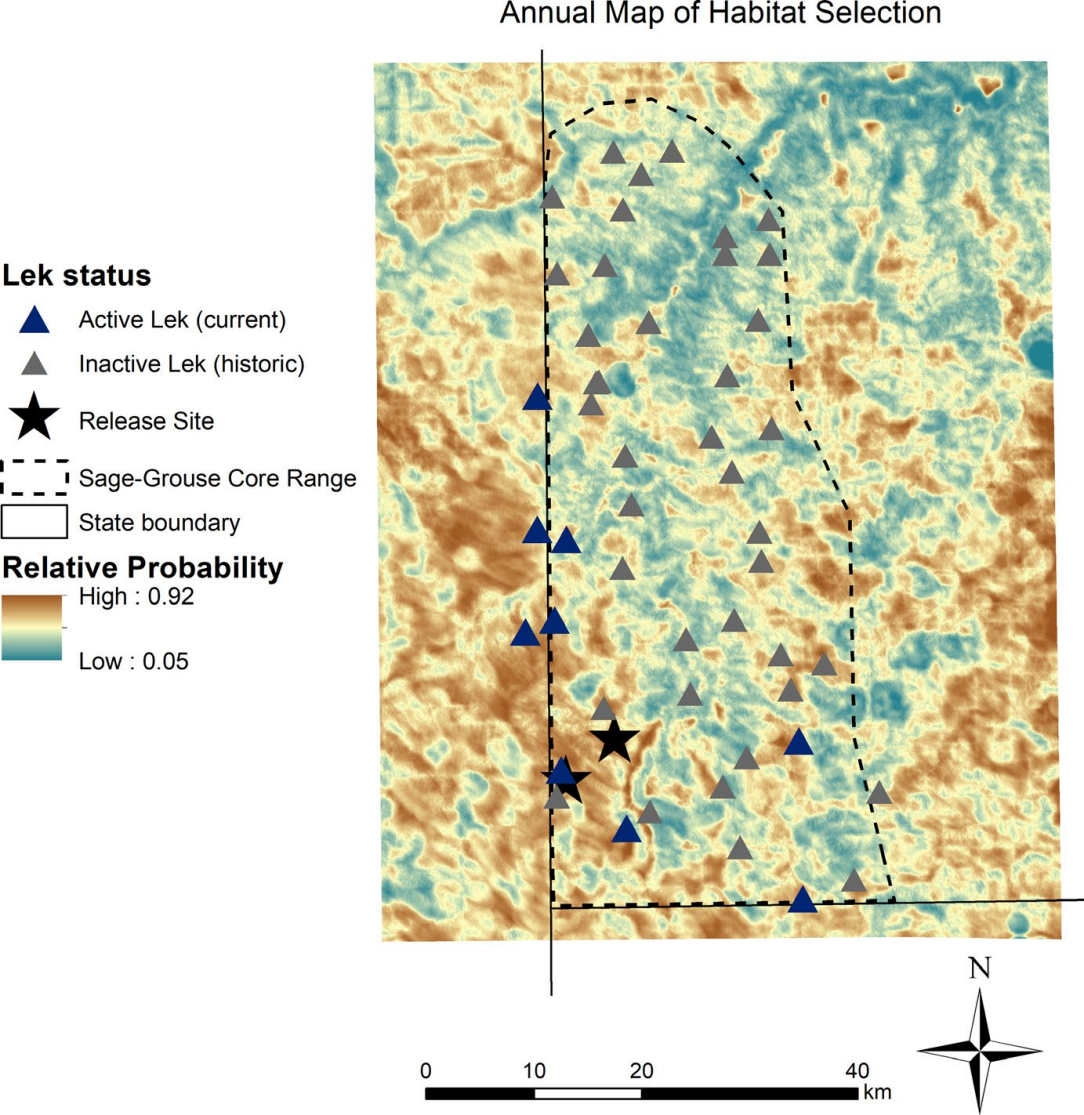
Annual composite map of predicted Greater sage‐grouse (*Centrocercus urophasianus*) habitat selection during 2005–2008 and 2017–2018 in southwestern North Dakota. The composite map was derived from the geometric mean of predicted seasonal habitat selection resource selection function surfaces of nesting, brood rearing, and summer nonbreeding seasons, respectively. Warmer color shades indicate greater relative probability of selection compared to cool color shaded areas

We observed the greatest loss of shrub cover and habitat immediately adjacent to the release site, where shrub cover was relatively dense during 1987 (Figure 6). Potential loss of habitat coincided with some of the areas indicated to be highly selected across seasons (Figure 7). We found variability in the loss and gain of habitat across different life stages at broader extents. However, greater losses of habitat overall were observed during the breeding season (nesting and brood‐rearing life stages) compared to nonbreeding summer months (Figure 6). Furthermore, during the breeding season, areas classified as high relative probability of selection typically experienced greatest losses of habitat since 1987. On the contrary, during summer, we found a positive relationship between relative probability of selection and habitat gains, on average.

## 4 DISCUSSION

We identified habitat relationships and developed spatially explicit predictions of habitat selection across different life‐history stages for translocated sage‐grouse to better understand which environmental factors and areas are currently supporting a remnant population on the fringe of their distributional range in southwestern North Dakota. Habitat requirements for nontranslocated sage‐grouse have been studied and synthesized in other areas of their range (see Connelly et al., 2000, 2011) and translocation actions that promote successful population restoration have been documented (see Wolfe et al., 1996). However, our study represents the first application of an RSF framework to spatially map seasonal habitat selection of sage‐grouse following a translocation to a new site. Importantly, we note that habitat selection patterns of translocated sage‐grouse were strongly tied to release site location, at least initially. Thus, selection of the release sites is likely to be critically important to future translocation success for sage‐grouse. In this study, release sites were based on historical habitat use patterns and lek site locations and thereby represented best available information. Following translocation, we found substantial variation across life stages in selection for shrub canopy cover, elevation, topographic roughness, distance to water, distance to roads, and mesic habitat variables. We provide managers with information about the relative influences of habitat factors which also may provide insight into changes in habitat across different life stages over the previous 30 years. These findings establish baseline habitat assessments in southwestern North Dakota, where the spatially explicit predictions can guide future studies that evaluate the quality and effectiveness of these habitats in promoting long‐term persistence and translocation success.

Changes in land use activities have altered the habitats of sage‐grouse within North Dakota (Larrucea & Brussard, 2008). As expected, translocated sage‐grouse selected areas with greater shrub cover than in available habitats at every life‐history stage in our study, but the importance of sagebrush shrub cover was most evidenced during the nesting period, similar to findings of Herman‐Brunson et al. (2009) and those from a nearby study area in South Dakota, where nesting sage‐grouse used relatively high amounts of sagebrush canopy cover (Kaczor et al., 2011). Although shrub cover was very influential, our study area consisted of typically lower cover values than recommendations published in sage‐grouse habitat guidelines (i.e., 15%–25%; Connelly et al., 2000; Hagen et al., 2007). Importantly, published guidelines were based on measurements conducted in the field at finer microhabitat scales (i.e., immediately around nests), whereas our results were derived from GIS‐level data collected from remote sensing applications at coarser spatial extents (*r* = 887 m). Thus, we expected lower values in our models, as cover was averaged across larger spatial areas. In addition, sagebrush cover and height are generally expected to be lower on the northeastern edge of sage‐grouse range than in other more mountainous areas of its interior range. Scale‐dependent processes are widely recognized in evaluations of sage‐grouse habitat (e.g., Coates, Brussee, et al., 2016; Coates, Casazza, et al., 2016; Fedy et al., 2014), and these nuances commonly warrant consideration by land managers.

Sagebrush shrub cover is vitally important to sage‐grouse populations, and our results agree with others (Schroeder et al., 2004) in recognizing the negative effects of sagebrush fragmentation and loss have on sage‐grouse populations, which is potentially especially pronounced on the fringe of the species range. Habitat limitations may impact success at restoring populations using translocation. The loss of shrub cover in North Dakota can be attributed to multiple factors, including conversion of grasslands to agricultural food crops, improper livestock grazing, energy development, and exurban expansion (Knick & Connelly, 2011). Selection for areas with greater shrub cover garnered strong support from our models at multiple life stages, indicating it may be imperative to first consider the relative loss of cover in initial release site assessments, especially considering typical translocation efforts occur during breeding spring months. For example, sagebrush loss was likely most significant for nesting and brood‐rearing activities, where cover was selected at relatively smaller scales and needed immediately at nest and brood locations for concealment, whereas during the nonbreeding summer months, patterns of selection were supported by data at broader spatial scales. Additionally, the linear effect associated with selection of shrub cover during the breeding months suggested that relatively dense cover appeared to be beneficial in this study area, whereas sage‐grouse prefer a mid‐range of shrub during nonbreeding summer months. Our study area typically comprised moderately dense shrub cover, so we likely could not detect nonlinear relationships that might otherwise be associated with higher shrub densities. In addition, the change in effect during summer is expected as sage‐grouse move into more mesic areas in search of water resources that are typically open (e.g., springs and seeps) as conditions become drier and warmer.

In addition to shrub cover, another consistently influential variable across all life stages was topographic roughness, specifically at the largest spatial scale (887 m). Thus, relatively flat areas measured at ~250 ha were preferred by translocated sage‐grouse, especially during the brood rearing and summer months. In southwest North Dakota, steeper and more rugged terrain often comprises exposed rock and sediment characteristic of the Badlands region, and offers minimal vegetation cover and/or water resources. Although relative selection of topographic characteristics by sage‐grouse varies throughout its distribution (e.g., Fedy et al., 2014; O’Neil et al., 2020; Walker et al., 2016), this topographic effect garnered substantial support from the data in North Dakota and may be a critical topographic component to consider in designing translocation projects. Importantly, other systems occurring at higher elevations may exhibit different relationships with topographic components, which also may vary depending on spatial scale of measurement.

We also observed variation in the effects of some environmental factors across life stages. For example, sage‐grouse showed greater avoidance for roads during the brood rearing and summer months than earlier in the nesting season, which was consistent with nontranslocated sage‐grouse (Fedy et al., 2014; Hagen et al., 2007). Roads are associated with many anthropogenic features, many of which have potential to impact sage‐grouse populations (Johnson et al., 2011). Oil and gas exploration require roads during construction and maintenance. Access roads are created to develop and maintain electric utility and distribution lines, which are also associated with oil wells, refineries, and other energy structures. Kohl et al. (2019) were unable to isolate the effects of power lines from the effects of roads on sage‐grouse habitat selection in Utah. Additionally, tall structures have also been described as a deterrent to sage‐grouse during various stages of their life history (Dinkins et al., 2014; Gibson et al., 2018; Hovick et al., 2014; Kohl et al., 2019) and are also typically associated with access roads. Although investigation of multiple types of anthropogenic infrastructures was beyond the scope of this study, our results indicated that sage‐grouse primarily avoided roads, which may serve as a proxy for other anthropogenic impacts on sage‐grouse habitats. As technologies in energy development advance in remote sagebrush environments, leading to expansion of road networks and additional habitat fragmentation, rescuing small populations of sage‐grouse that have experienced loss of suitable habitat will inevitably become more challenging. Additionally, our study did not investigate impacts of roads or other anthropogenic structure on population vital rates. Thus, further research that evaluates relationships between anthropogenic features and postrelease performance of sage‐grouse would be beneficial to pretranslocation decision analysis while also identifying mechanisms that prevent populations from recovering.

Although translocations do not generally occur during the summer season, our results for brooding and nonbrooding habitat selection during this period provide important information about potential habitat requirements beyond the breeding season. We found that during summer months, translocated sage‐grouse preferred mesic areas but avoided waterways, which was similar to nontranslocated sage‐grouse elsewhere (Fedy et al., 2014). Perhaps, this was because waterways represent linear riparian sites that coincide with a higher risk of predation, as opposed to irregularly shaped mesic sites in the form of wet meadows that consist of sagebrush integrated with grasses and forbs. Notably, the effect of proximity to the pseudo‐lek release location was not observed during summer, unlike those effects observed during nesting and brood‐rearing phases. This should be expected as all sage‐grouse were translocated to the release area immediately prior to nesting or during the brood‐rearing period. Because relatively more time has elapsed between the release dates and summer season, sage‐grouse have more opportunity to explore the area and select habitats independent of release site location. Thus, the diminishing effect of release location from nesting to summer seasons underscore the importance of accounting for release site in the RSF models during breeding months. In addition, spatial dependence occurring in association with the release site can have a particularly strong influence on nest site selection and can lead to misleading statistical results if ignored in the modeling phase. Because nest site location then influences habitat availability for broods, the release site location can have habitat selection effects that continue through the summer for this important segment of the population.

In conclusion, our findings d how translocated sage‐grouse used a novel environment and serve as baseline information for assessment of seasonal habitat quality at release locations occurring at relatively large spatial scales. Our spatial predictions imply areas of high versus low relative use, which is often assumed to coincide with gradients of habitat quality. However, future evaluations are needed to inform the population's performance as a response to the same habitat characteristics that are being selected or avoided across life stages, because habitat selection may not align with population performance, especially when individuals have imperfect knowledge of their environment (Cutting et al., 2019; O’Neil et al., 2020; Robertson et al., 2013). Information on population performance is needed to help guide placement of future release sites. For example, changing the release location of translocated sharp‐tailed grouse to an area with more suitable breeding habitats substantially reduced dispersal from the release site and promoted localization in Nevada (Coates et al., 2006). Notably, sage‐grouse have continued to occupy areas where sagebrush cover has been conserved over time within our study area. Increased efforts to conserve the remaining sagebrush cover and to restore sagebrush communities within our study area are likely key to successful restoration and future persistence of sage‐grouse within North Dakota. Because selection of resources is directly tied to the release site locations that are chosen by researchers or managers, an important consideration when performing translocation is that other augmented populations may provide different outcomes. Although the greater sage‐grouse is currently at risk of extirpation in North Dakota, current information suggests that available habitat was, at minimum, adequate to support fidelity and space use by translocated individuals across breeding and summer life stages near release sites. Future studies on population performance and potential restoration of historical shrub communities could further inform and/or increase the extent of suitable habitat, thereby promoting long‐term sustainability of the greater sage‐grouse population in North Dakota.

## CONFLICT OF INTEREST

The authors declare no conflicts of interest. Any use of trade, firm, or product names is for descriptive purposes only and does not imply endorsement by the U.S. Government.

## AUTHOR CONTRIBUTION

**Kade D. Lazenby:** Conceptualization (equal); Data curation (lead); Formal analysis (lead); Investigation (equal); Methodology (equal); Resources (equal); Validation (equal); Visualization (equal); Writing‐original draft (lead); Writing‐review & editing (equal). **Peter S Coates:** Conceptualization (supporting); Data curation (supporting); Formal analysis (supporting); Funding acquisition (lead); Investigation (supporting); Methodology (supporting); Project administration (lead); Resources (equal); Supervision (supporting); Validation (equal); Writing‐original draft (supporting); Writing‐review & editing (lead). **Shawn T. O'Neil:** Conceptualization (supporting); Formal analysis (equal); Investigation (supporting); Methodology (equal); Validation (equal); Visualization (equal); Writing‐original draft (supporting); Writing‐review & editing (equal). **Michel Kohl:** Conceptualization (supporting); Data curation (supporting); Formal analysis (supporting); Investigation (supporting); Methodology (equal); Validation (supporting); Visualization (supporting); Writing‐original draft (supporting); Writing‐review & editing (supporting). **David Dahlgren:** Conceptualization (equal); Data curation (supporting); Formal analysis (supporting); Funding acquisition (equal); Investigation (supporting); Methodology (supporting); Project administration (lead); Resources (equal); Supervision (lead); Validation (supporting); Visualization (supporting); Writing‐original draft (supporting); Writing‐review & editing (supporting).

## Supporting information

Supplementary Material

## Data Availability

Geospatial layers and data supporting the results of this manuscript are available for public download at the USGS *ScienceBase* website and digital repository (https://doi.org/10.5066/P91GQXVE; Coates et al., 2021).

## 1

Tables of scale selection parameters and resource selection coefficients from nesting, brood rearing, and summer habitat selection analysis.

**TABLE A1:** Scale selection parameters from a Bayesian latent indicator scale selection (Stuber et al., 2017) model of Greater sage‐grouse (*Centrocercus urophasianus*) nest site and brood locations, where parameters indicate probabilistic support for varying neighborhood radius size characterizations of shrub cover and topographic roughness (60, 331, and 887 m, respectively)

Scale	Nest model probability	Brood model probability
Shrub cover (*r* = 60)	0.270	0.150
Shrub cover (*r* = 331)	0.252	0.399
Shrub cover (*r* = 887)	0.478	0.451
Roughness (*r* = 60)	0.065	0.000
Roughness (*r* = 331)	0.068	0.126
Roughness (*r* = 887)	0.867	0.874

Note

Scale selection was implemented for resource selection models of greater sage‐grouse nests and broods within southwestern North Dakota, USA.

**TABLE A2:** Posterior distribution means, medians, standard deviations, and percentiles of coefficient estimates from resource selection models of greater sage‐grouse (*Centrocercus urophasianus*) nests, broods, and nonbreeding summer locations contrasted with random available locations within North Dakota, USA

Life stage	Parameter	Mean	*SD*	2.5th	50th	97.5th	*f*	$\hat{r}$
Nesting	Intercept	−2.110	0.257	−0.264	−2.100	−1.636	1.000	1.001
	Aspect	0.035	0.175	−0.314	0.029	0.391	0.575	1.000
	Elevation	−0.133	0.221	−0.626	−0.108	0.257	0.722	1.001
	Proximity to water	−0.092	0.185	−0.498	−0.074	0.245	0.679	1.000
	Proximity to mesic	0.065	0.179	−0.279	0.053	0.441	0.638	1.000
	Proximity to road	−0.048	0.175	−0.423	−0.039	0.295	0.601	1.000
	Proximity to release location	1.309	0.255	0.840	1.297	1.844	1.000	1.000
	Shrub cover (*r* = 887 m)	0.235	0.213	−0.136	0.221	0.684	0.878	1.000
	Roughness (*r* = 887 m)	−0.577	0.267	−1.121	−0.567	−0.072	0.991	1.000
Brood rearing	Intercept	−2.285	0.663	−3.438	−2.241	−1.457	0.998	1.076
	Aspect	0.216	0.175	−0.096	0.207	0.579	0.902	1.000
	Elevation	0.160	0.206	−0.207	0.140	0.603	0.775	1.000
	Proximity to water	−0.083	0.161	−0.422	−0.073	0.218	0.693	1.000
	Proximity to mesic	−0.141	0.171	−0.493	−0.133	0.182	0.799	1.000
	Proximity to road	−0.734	0.200	−1.143	−0.728	−0.356	1.000	1.000
	Proximity to release location	0.395	0.179	0.066	0.387	0.769	0.994	1.000
	Shrub cover (*r* = 887 m)	0.388	0.240	−0.028	0.374	0.890	0.960	1.000
	Roughness (*r* = 887 m)	−1.001	0.247	−1.497	−0.993	−0.544	1.000	1.000
Summer, nonbreeding	Intercept	−1.854	0.211	−2.307	−1.843	−1.453	1.000	1.000
	Aspect	0.064	0.051	−0.033	0.064	0.164	0.893	1.000
	Elevation	−0.490	0.068	−0.623	−0.490	−0.357	1.000	1.000
	Proximity to water	−0.597	0.057	−0.710	−0.596	−0.488	1.000	1.001
	Proximity to mesic	0.206	0.051	0.107	0.206	0.306	1.000	1.000
	Proximity to road	−0.664	0.054	−0.772	−0.664	−0.56	1.000	1.000
	Proximity to release location	0.066	0.047	−0.027	0.066	0.158	0.922	1.000
	Shrub cover (*r* = 1,503 m)	1.311	0.108	1.103	1.310	1.522	1.000	1.000
	Roughness (*r* = 767 m)	−0.392	0.064	−1.049	−0.918	−0.796	1.000	1.001
	Shrub cover (quadratic; *r* = 1,503 m)	−0.715	0.069	−0.853	−0.714	−0.584	1.000	1.000

Note

The *f*‐statistic indicates the proportion of the distribution having the same sign as the mean coefficient estimate, and the $\hat{r}$ statistic indicates model convergence for values <1.1. Nest locations were collected during 2005–2008 and 2017–2018.
