# Supplementary material for: Nesting, brood rearing, and summer habitat selection by translocated greater sage‐grouse in North Dakota, USA
Source: Ecol Evol. 2021 Feb 19;11(6):2741–60. doi: 10.1002/ece3.7228 (PMC7981223; doi:10.1002/ece3.7228)
Supplement: Supplementary file 1 — Supplementary Material [file ECE3-11-2741-s001.docx]

**APPENDIX 1**. Tables of scale selection parameters and resource selection coefficients from nesting, brood-rearing, and summer habitat selection analysis.

Table A1. Scale selection parameters from a Bayesian latent indicator scale selection (Stuber et al. 2017) model of Greater sage-grouse (*Centrocercus urophasianus*) nest site and brood locations, where parameters indicate probabilistic support for varying neighborhood radius size characterizations of shrub cover and topographic roughness (60 m, 331 m, and 887 m, respectively). Scale selection was implemented for resource selection models of greater sage-grouse nests and broods within southwestern North Dakota, USA.

| Scale | Nest model probability | Brood model probability |
| --- | --- | --- |
| Shrub cover (r=60) | 0.270 | 0.150 |
| Shrub cover (r=331) | 0.252 | 0.399 |
| **Shrub cover (r=887)** | **0.478** | **0.451** |
| Roughness (r=60) | 0.065 | 0.000 |
| Roughness (r=331) | 0.068 | 0.126 |
| **Roughness (r=887)** | **0.867** | **0.874** |

Table A2. Posterior distribution means, medians, standard deviations, and percentiles of coefficient estimates from resource selection models of greater sage-grouse (*Centrocercus urophasianus*) nests, broods, and non-breeding summer locations contrasted with random available locations within North Dakota, USA. The f-statistic indicates the proportion of the distribution having the same sign as the mean coefficient estimate, and the $\hat{r}$ statistic indicates model convergence for values <1.1. Nest locations were collected during 2005-2008 and 2017-2018.

| Life stage | Parameter | Mean | SD | 2.5^th^ | 50^th^ | 97.5^th^ | f | $\hat{r}$ |
| --- | --- | --- | --- | --- | --- | --- | --- | --- |
| Nesting | Intercept | -2.110 | 0.257 | -0.264 | -2.100 | -1.636 | 1.000 | 1.001 |
|  | Aspect | 0.035 | 0.175 | -0.314 | 0.029 | 0.391 | 0.575 | 1.000 |
|  | Elevation | -0.133 | 0.221 | -0.626 | -0.108 | 0.257 | 0.722 | 1.001 |
|  | Proximity to water | -0.092 | 0.185 | -0.498 | -0.074 | 0.245 | 0.679 | 1.000 |
|  | Proximity to mesic | 0.065 | 0.179 | -0.279 | 0.053 | 0.441 | 0.638 | 1.000 |
|  | Proximity to road | -0.048 | 0.175 | -0.423 | -0.039 | 0.295 | 0.601 | 1.000 |
|  | Proximity to release location | 1.309 | 0.255 | 0.840 | 1.297 | 1.844 | 1.000 | 1.000 |
|  | Shrub cover (r=887 m) | 0.235 | 0.213 | -0.136 | 0.221 | 0.684 | 0.878 | 1.000 |
|  | Roughness  (r=887 m) | -0.577 | 0.267 | -1.121 | -0.567 | -0.072 | 0.991 | 1.000 |
| Brood-rearing | Intercept | -2.285 | 0.663 | -3.438 | -2.241 | -1.457 | 0.998 | 1.076 |
|  | Aspect | 0.216 | 0.175 | -0.096 | 0.207 | 0.579 | 0.902 | 1.000 |
|  | Elevation | 0.160 | 0.206 | -0.207 | 0.140 | 0.603 | 0.775 | 1.000 |
|  | Proximity to water | -0.083 | 0.161 | -0.422 | -0.073 | 0.218 | 0.693 | 1.000 |
|  | Proximity to mesic | -0.141 | 0.171 | -0.493 | -0.133 | 0.182 | 0.799 | 1.000 |
|  | Proximity to road | -0.734 | 0.200 | -1.143 | -0.728 | -0.356 | 1.000 | 1.000 |
|  | Proximity to release location | 0.395 | 0.179 | 0.066 | 0.387 | 0.769 | 0.994 | 1.000 |
|  | Shrub cover (r=887 m) | 0.388 | 0.240 | -0.028 | 0.374 | 0.890 | 0.960 | 1.000 |
|  | Roughness  (r=887 m) | -1.001 | 0.247 | -1.497 | -0.993 | -0.544 | 1.000 | 1.000 |
| Summer, non-breeding | Intercept | -1.854 | 0.211 | -2.307 | -1.843 | -1.453 | 1.000 | 1.000 |
|  | Aspect | 0.064 | 0.051 | -0.033 | 0.064 | 0.164 | 0.893 | 1.000 |
|  | Elevation | -0.490 | 0.068 | -0.623 | -0.490 | -0.357 | 1.000 | 1.000 |
|  | Proximity to water | -0.597 | 0.057 | -0.710 | -0.596 | -0.488 | 1.000 | 1.001 |
|  | Proximity to mesic | 0.206 | 0.051 | 0.107 | 0.206 | 0.306 | 1.000 | 1.000 |
|  | Proximity to road | -0.664 | 0.054 | -0.772 | -0.664 | -0.56 | 1.000 | 1.000 |
|  | Proximity to release location | 0.066 | 0.047 | -0.027 | 0.066 | 0.158 | 0.922 | 1.000 |
|  | Shrub cover (r=1503 m) | 1.311 | 0.108 | 1.103 | 1.310 | 1.522 | 1.000 | 1.000 |
|  | Roughness  (r=767 m) | -0.392 | 0.064 | -1.049 | -0.918 | -0.796 | 1.000 | 1.001 |
|  | Shrub cover (quadratic; r=1503 m) | -0.715 | 0.069 | -0.853 | -0.714 | -0.584 | 1.000 | 1.000 |
